# The Long-Term Results of Electrochemotherapy in the Treatment of Patients with Locoregionally Advanced, Unresectable Melanoma

**DOI:** 10.3390/jcm13133705

**Published:** 2024-06-25

**Authors:** Marcin Zdzienicki, Marcin Ziętek, Maria Krotewicz, Agnieszka Ewert-Krzemieniewska, Piotr Rutkowski

**Affiliations:** 1 Department of Soft Tissue and Bone Sarcomas and Melanomas, Maria Sklodowska-Curie National Research Institute of Oncology in Warsaw, 02-791 Warszawa, Poland; 2 Department of Oncology, Wroclaw Medical University, 53-413 Wrocław, Poland; marcin.zietek@dcopih.pl; 3 Department of Surgical Oncology, Lower Silesian Oncology, Pulmonology and Hematology Center, 53-413 Wrocław, Poland

**Keywords:** skin melanoma in-transit metastases, locoregional treatment, electrochemotherapy

## Abstract

**Background/Objectives**: Despite observing progress in recent years in the treatment of patients with advanced melanoma, the optimal management of locoregional recurrence has not been determined. Various methods are used to treat this group of patients. One of these methods is electrochemotherapy. The present study presents the distant results in treating patients with the locoregional recurrence of melanoma, using the technique of electrochemotherapy. **Methods:** This study includes a retrospective analysis of 88 patients’ data with locoregional melanoma recurrence, treated with electrochemotherapy (ECT) between 2010 and 2023, in two reference centers. **Results:** Approximately 80% of patients responded to the ECT treatment, achieving partial or complete remission. In a multivariate analysis, statistically significant longer overall survival was found in the group of patients who achieved complete remission after ECT and were treated with immunotherapy. **Discussion:** The results may suggest the existence of synergy between ECT and immunotherapy. However, confirmation of this fact requires further prospective studies that will also establish the role of ECT in the combination treatment of patients with locoregional recurrence of melanoma.

## 1. Introduction

Despite the progress observed for many years in the treatment of patients with advanced melanoma, many problems remain unsolved [1,2]. One of the problems is the management of the locoregional recurrence of melanoma. This category includes satellite foci arising around the scar (up to 2 cm) from the resection of the primary tumor or in-transit metastases. In-transit lesions are defined as metastases, appearing between the primary foci and the regional lymphatic basin. Locoregional recurrences fall into the clinical stage III (B-D) of melanoma [3]. The frequency of this type of locoregional recurrence is estimated at 5 to 10% of all melanoma patients [4,5]. Many methods are in use in patients with local relapses or in-transit metastases. For resectable lesions, surgical treatment involving the local excision of the nodules remains the option of choice. For unresectable lesions, one of several local or regional treatment techniques can be used. These include intratumoral therapies (like T-Vec, electrochemotherapy-ECT) or regional treatment (e.g., isolated limb perfusion-ILP) [6,7,8]. Unfortunately, none of the techniques mentioned gives satisfactory long-term results [9]. These patients, despite treatment, are at a very high risk of another recurrence of the disease. These risks, combined with other unfavorable prognostic factors (advanced primary tumors, coexisting regional lymph node metastases), can reach up to 90% in some cases [10]. The combination of available methods seems to be a promising solution to the problem. However, there is insufficient evidence, in the form of multicenter randomized trials, to develop standards for the management of multiple/advanced locoregional melanoma recurrence.

One of the methods mentioned, applicable to this group of patients, is ECT [6,11].

This method uses the phenomenon of reversible electroporation. It involves the temporary destabilization of the cell membrane under the influence of an electric field with appropriately selected parameters. This destabilization allows free diffusion of molecules between the extracellular fluid and the cytosol, bypassing the natural transmembrane transport mechanisms. In the case of electrochemotherapy, we use this phenomenon to increase the intracellular concentration of the chemotherapy drug previously administered intravenously or intratumorally [12].

The management of patients with in-transit/subcutaneous metastases is not well established, and ECT is the proposed therapy method, making real-world evidence important for this technique [13,14].

This paper aims to present the experience of two Polish reference centers in the treatment of locoregional melanoma recurrences with electrochemotherapy and attempt to evaluate the long-term results of this treatment.

## 2. Materials and Methods

The presented work includes retrospective data analysis from 88 consecutive patients with locoregional relapses of melanoma—unresectable local relapses or metastases to the skin, subcutaneous tissue (in transit), or lymph nodes. These patients were treated between 2010 and 2023 using electrochemotherapy. Seventy-seven patients underwent treatment at the Department of Soft Tissue and Bone Sarcomas and Melanomas of Maria Sklodowska-Curie National Research Institute of Oncology in Warsaw. Eleven patients were treated in the 2nd Department of Surgical Oncology of the Lower Silesian Center for Oncology, Pulmonology and Hematology in Wrocław. This study included 36 men and 52 women. The ages of the patients ranged from 33 to 93 years. The median age was 67 years. Lesions subjected to electrochemotherapy were located on the lower limb in 75 patients (85%), on the upper limb in 8 patients (9%), and on the trunk in 5 patients (6%). The characteristics of the patients included in the study at both participating centers are presented in Table 1.

**Figure 1:**
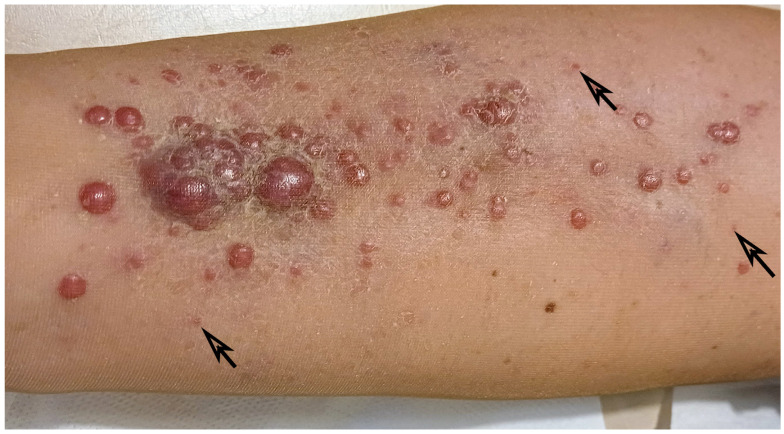
The picture presents an example of in-transit melanoma metastases on the lower legs’ skin. Arrows indicate small (less than 1 mm in diameter) skin lesions, difficult to interpret—most likely also in-transit nodules.

**Figure 2:**
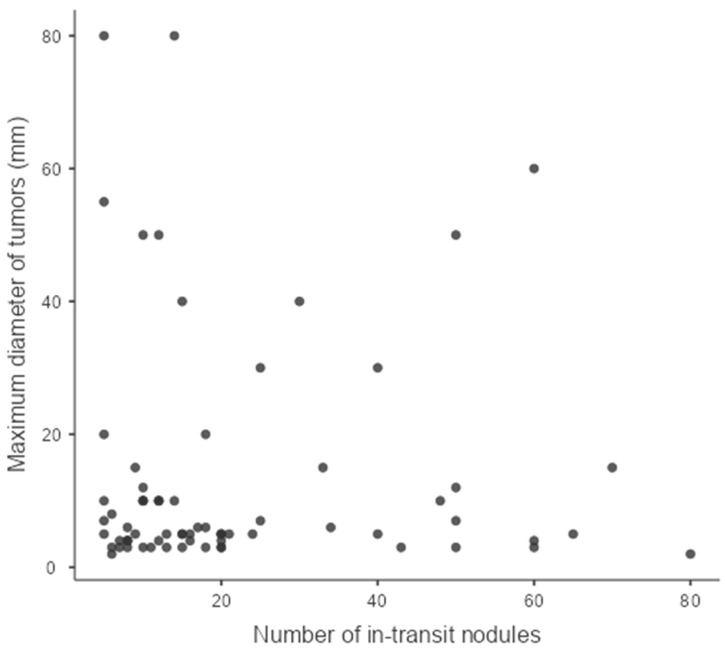
Graph showing the distribution of size and number of nodules in the analyzed group.

In all patients, the indication for ECT was unresectable locoregional relapse of melanoma. The reason for non-resectability was the number of lesions (over 5) or their topography (the area affected by in-transit metastases prevented radical surgical treatment) as assessed by a multidisciplinary team with at least two experienced oncological surgeons. The number of lesions treated in one patient ranged from 5 to over 60 nodules. In patients with greater numbers of lesions, determining the exact number was impossible due to their often-confluent nature (Figure 1). The distribution of size and number of nodules treated with ECT is presented in Figure 2.

Only a low percentage of patients in the analyzed group received systemic treatment before electrochemotherapy. Eight patients (9%) treated before 2013 received systemic chemotherapy based on dacarbazine. Among the patients treated after 2013, 2 (3.4%) had received immunotherapy before ECT. 

In all patients, electrochemotherapy was performed with intravenous administration of bleomycin at a dose of 15,000 IU per square meter of body surface area. In all cases, electroporation was performed with Cliniporator2^®^ equipment (IGEA S.p.A. Via Parmenide, 10/A 41012 Carpi (MO)-Italia). Due to the topography of the lesions, the most frequently used electrode for electroporation was a 20 mm long electrode with a hexagonal needle arrangement. After the treatment, the patients were strictly observed. In the case of the recurrence or progression of the disease, patients were qualified for further treatment. The 11 patients underwent the second electrochemotherapy procedure due to locoregional relapse. These treatments were repeated 2 to 28 months (median 6 months) after the initial treatment.

The follow-up time in the study group ranged from 1 to 125 months, and the median follow-up time was 13 months. 

Overall survival (OS) and progression-free survival (PFS) curves were calculated using the Kaplan–Meier method. Survival differences between the groups were assessed using the log-rank test. Multivariable Cox regression analyses were performed on factors influencing OS and PFS. All calculations were performed with Jamovi software (The Jamovi project (2023). Jamovi (Version 2.4) Retrieved from https://www.jamovi.org (accessed on 31 December 2023)). This is an open-source statistical platform, based on R-soft (R Core Team (2022). R: A Language and environment for statistical computing (Version 4.1). Retrieved from https://cran.r-project.org (accessed on 31 December 2023) (R packages retrieved from CRAN snapshot 7 April 2023)).

## 3. Results

Determining the local response to treatment in in-transit metastatic melanoma is not straightforward, and it is mainly based on clinical evaluations. In patients who underwent ECT, the lesions were assessed at intervals of 3 weeks after treatment. Usually, the full effect of treatment was observed after 6 to 9 weeks. If there were no changes up to 12 weeks after the procedure, the response was defined as stabilization. 

In an evaluation of the preliminary result of ECT, 40 patients (45%) showed complete remission of the treated lesions. A partial response was observed in 31 patients (35%). Three patients had disease stabilization; four others revealed a locoregional progression at the first evaluation. The response to ECT was not assessed in 10 patients. To summarize, 80% of patients responded to the ECT treatment. 

Due to the progression of the disease, 35 patients in the analyzed group received systemic chemotherapy (based on dacarbazine). Thirty-three patients were treated with immunotherapy (anti-CTLA-4 or/and anti-PD-1). Nineteen patients received BRAF inhibitors (iBRAF) or the iBRAF/iMEK combination. In the entire study group, only 25 (28%) patients did not receive any systemic treatment. Sixteen patients (18%) were treated exclusively with dacarbazine-based chemotherapy. The remaining patients received various types of systemic therapy depending on the local and general condition. 

The distribution of different systemic treatments in the study group is shown in Table 2.

**Figure 3:**
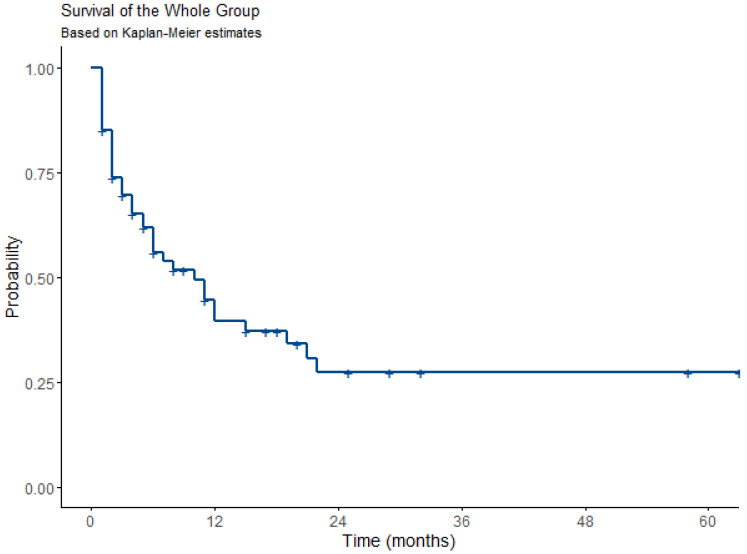
The Kaplan–Meier plot of overall survival in the entire group.

The study group was analyzed in terms of overall survival (OS) and progression-free survival (PFS) after the ECT procedure. We also made an attempt to identify factors influencing survival times. The overall survival rate in the study group was 70% after one year, 51% after three years, and 28.5% after five years. The median survival time was 37 months (18–58 months, 95% CI). These data are presented in Table 3 and Figure 3, respectively.

About 24% of patients had only locoregional progression in the form of the next wave of in-transit lesions. In another 27% of patients, distant metastases were revealed during the follow-up period. About 16.7% of patients relapsed in both ways (local progression and distant metastases). In the vast majority of patients, distant lesions were located in the central nervous system. 

**Figure 4:**
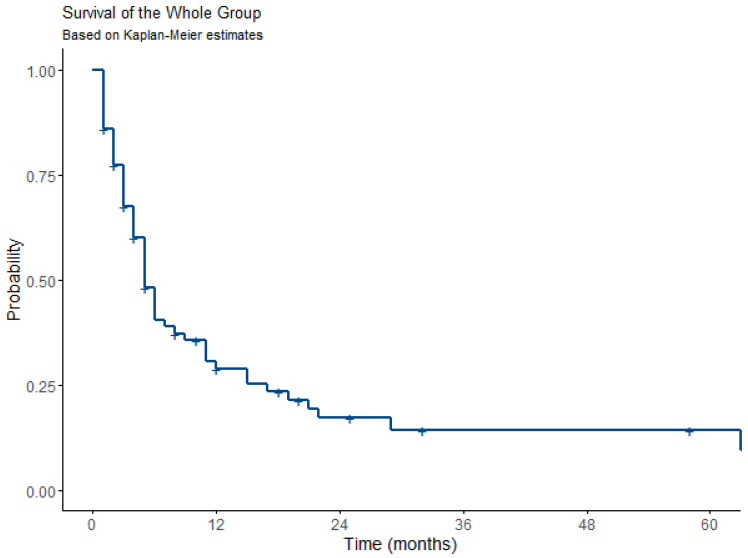
Kaplan–Meier plot of progression-free survival.

In the Kaplan–Meier survival estimation, the median progression-free survival was 5 months (4–9 months, 95% CI). Progression-free survival (PFS) rates were 28.8% after one year and 14.3% after 3 and 5 years. These data are presented in Table 4 and Figure 4, respectively.

Then, we assessed the impact of other treatments used with electrochemotherapy on overall survival and progression-free survival. For this purpose, we performed appropriate multivariate analyses. In multivariate analysis, none of the treatment methods used showed a statistically significant impact on overall survival. In addition, we examined whether the patient’s age or gender had an impact on distant treatment outcomes. We found no statistically significant effect of these factors on OS or PFS.

When assessing the impact of other treatment methods on PFS survival, we revealed that patients who received dacarbazine-based chemotherapy in addition to electrochemotherapy had significantly worse outcomes. The result was statistically significant. The results of multivariate analyses are presented in Table 5 and Table 6.

We decided to perform a similar multivariate analysis, including only patients whose first ECT treatment led to complete remission. This analysis showed once again a worse prognosis in patients in whom ECT was combined with systemic chemotherapy. The analysis also illustrated a better prognosis in patients who received ECT and systemic immunotherapy. These results were also statistically significant, as shown in Table 7. 

**Figure 5:**
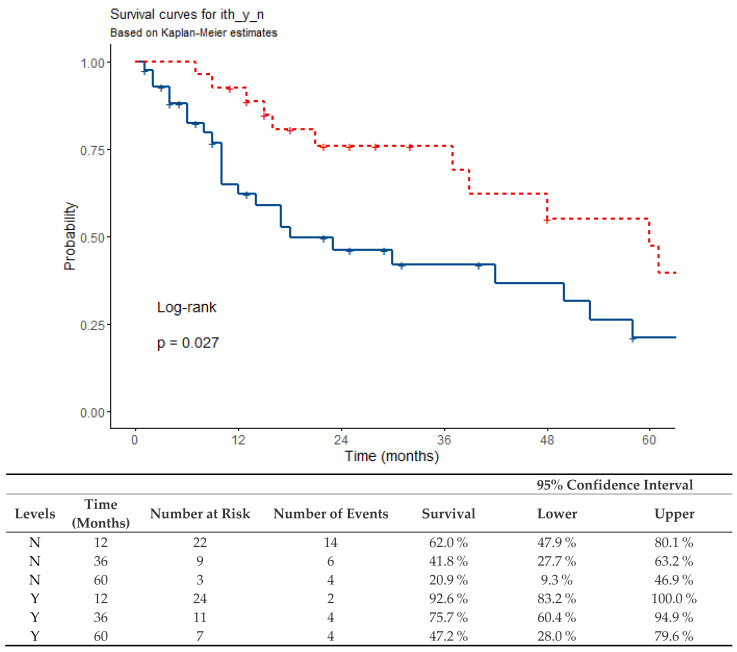
Survival plot and table for CR ECT patients receiving or not (Y/N) systemic immunotherapy (red dashed curve = Y, blue curve = N).

Overall survival was 62% at one year, 42% at three years, and 21% at five years in patients who did not receive immunotherapy (median 18 months (12–58 months, 95% CI)). For patients who received systemic immunotherapy, these survival rates were 92.6% after one year, 75.7% after three years, and 47% after five years, respectively (median survival, in this case, was 60 months (from 39 months; the upper limit was not reached for 95% CI)) The survival plot of patients treated or not with immunotherapy after complete remission achieved with ECT is presented in Figure 5.

The multivariate analysis of PFS did not reveal any new relationships in the patients who achieved complete remission after ECT. This analysis still showed a negative correlation between progression-free survival and systemic chemotherapy in these patients. The results of this analysis are shown in Table 8.

The PFS in patients with a complete response after ECT is comparable to the results obtained in the entire study group. Patients additionally treated with immunotherapy achieved slightly higher PFS than patients without treatment. This difference is not statistically significant. These relationships are included in Table 9.

## 4. Discussion

Electrochemotherapy is a highly effective method of destroying superficially located tumor foci. The overall response rate reported in the literature reaches 80–90% [15]. In the study group, we achieved complete remissions in 45% of cases, while other authors reported complete remissions in approximately 60% of patients. This difference may be because of the subjective assessment of the response to ECT. Sometimes, melanin deposits remaining at the electroporation site may be interpreted as a residual melanoma lesion. Nevertheless, our observed overall response rate is consistent with the literature data [15,16,17].

Our patients’ OS and PFS are lower than the currently expected results of treatment of patients with advanced melanoma using modern systemic therapies. This difference is most likely because some patients whose data were analyzed started treatment when modern therapies were not yet available. Almost 1/5 of patients with indications for systemic treatment received only dacarbazine-based chemotherapy. Nevertheless, long-term benefits in terms of lack of progression after ECT were observed in about 20% of patients.

The low effectiveness of standard chemotherapy in patients with advanced melanoma was beyond doubt. The results we presented confirm the lack of benefits of this type of treatment in the combined treatment of patients with locoregionally advanced melanoma [18].

Accurate assessment of the impact of systemic immunotherapy on the treatment outcomes of patients with locoregional melanoma recurrence is not easy. The available literature only presents the results of retrospective analyses. In 2022, Holberg et al. presented the results of the treatment of 287 patients with diagnosed in-transit metastases. These patients were treated at 21 institutions with systemic immunotherapy with PD-1 or CTLA-4 inhibitors or a combination of both. The median PFS was 10 months. The PFS rate was 48% after one year, 33% after two years, and 18% after five years. The melanoma-specific overall survival was 95% at one year, 83% at two years, and 71% at five years, respectively. Median survival was not assessed [19].

In a similar report published in 2020, Nan Tie presented the results of the treatment of 54 patients from three Australian centers. The median PFS obtained in this study was 11.7 months. The PFS was 48% at one year and 39% at two years. Overall survival was 85% at one year and 63% at two years. In this study, the median OS was also not reached [20]. On the other hand, reliable results of treatment of advanced melanoma with immunotherapy are available in the world literature. Randomized phase 3 multicenter trials using antiPD-1 revealed median survival rates of 30–40 months. In patients treated with anti-CTLA-4, the median survival rate was 15–20 months. The percentage of OS for anti-PD-1 therapy was about 50% after two years and about 45% after five years. With anti-CTLA-4 treatment, these percentages were about 40% and 26%, respectively [21,22,23].

In the study we present, the median survival is higher than that obtained in studies with immunotherapy. The five-year OS rate in our group is only slightly higher than that obtained in phase III trials. It should be noted that in the mentioned phase III trials, clinically unresectable stage III patients made up a relatively small percentage of treated patients. The majority of patients qualified for immunotherapy under these protocols were in stage IV. Therefore, a comparison of these studies with our results is not possible. These results, however, may suggest that ECT delays disease progression, without affecting OS. A definitive answer to this question is only possible through a prospective clinical trial.

Our study failed to demonstrate the effect of systemic immunotherapy on PFS in patients after local treatment of loco-regional melanoma recurrence using ECT. However, we revealed that combining ECT with systemic immunotherapy gives the best results in terms of OS reaching a rate of 50% at 5 years. 

However, taking into account the literature cited above, only systemic immunotherapy may be responsible for improving the results in this subgroup of patients. 

The work we present is not the first attempt to evaluate the association of ECT with other systemic treatments. There are reports presenting the results of retrospective analyses of the combination of ECT with immunotherapy. In 2015, Mozzillo et al. presented a retrospective evaluation of the treatment outcomes of 15 patients who underwent ECT during anti-CTLA-4 treatment. Patients achieved a 67% local response rate, and treatment tolerability was consistent with the safety profile of Ipilimumab. In this study, long-term outcomes were not assessed [24].

In 2016, Heppt et al. presented the results of treating 33 patients, combining immunotherapy and ECT. The analysis revealed the prolongation of median OS time in patients treated with immunotherapy in combination with ECT [25]. In 2021, Campana et al. presented a retrospective analysis of the results of treating 130 patients with ECT, anti-PD-1, or a combination of both modalities. This combination was superior to treatment with anti-PD-1 alone in terms of PFS and OS after two years [26].

A weakness of the cited studies and the present paper is their retrospective nature. Nevertheless, both the cited studies and our analysis may suggest the existence of synergy between electrochemotherapy and systemic immunotherapy. 

It was postulated, among other things, that ECT, destroying melanoma cells, releases tumor antigens, which thus become available to immunocompetent cells. This may improve the effectiveness of systemic immunotherapy [27,28].

The previously postulated similar effect of T-VEC, which is currently the standard treatment for local in-transit lesions, has not been confirmed. The advantage of combination treatment with T-VEC and pembrolizumab over pembrolizumab monotherapy has not been demonstrated [29].

## 5. Conclusions

Our study confirms that electrochemotherapy is an effective method of local control in locoregional recurrences of cutaneous melanoma. It seems that as a stand-alone method, it has little impact on long-term survival rates. However, the presented data suggest the existence of synergy between ECT and systemic immunotherapy. The confirmation of this effect and determining the place of electrochemotherapy in the combined treatment regimen for melanoma patients require further prospective studies.

## Figures and Tables

**Table 1:** Characteristics of the patients included in the study.

	Warszawa	Wrocław
Sex		
Women	49	6
Men	28	5
Age		
Range (years)	33–89	39–93
Median (years)	67	71
Location of the treated lesions		
Upper limb	6	2
Trunk	5	0
Lower limb	66	9

**Table 2:** Size of patient groups treated with different combinations of systemic therapies.

Type of Combination	Number of Patients Treated
Chemotherapy alone	16
Chemotherapy and immunotherapy	14
Chemotherapy and iBRAF (or iBRAK/iMEK)	2
Immunotherapy alone (anti CTLA4 or anti PD-1)	10
Immunotherapy and iBRAF (or iBRAF/iMEK)	6
iBRAf (or iBRAF/iMEK) alone	6
Chemotherapy and immunotherapy and iBRAF (or iBRAF/iMEK)	3

**Table 3:** Overall survival in the entire group after 1, 3, and 5 years, respectively.

				95% Confidence Interval	
Time of OS (Months)	Number at Risk	Number of Events	Survival	Lower	Upper
12	48	23	69.9%	60.3%	81.1%
36	20	11	50.8%	39.8%	64.9%
60	10	8	28.5%	17.7%	46.0%

**Table 4:** Progression-free survival after ECT in the entire group.

			95% Confidence Interval		
Time of PFS (Months)	Number at Risk	Number of Events	Survival	Lower	Upper
12	18	51	28.8 %	19.7 %	42.1 %
36	4	7	14.3 %	7.3 %	28.0 %
60	3	0	14.3 %	7.3 %	28.0 %

**Table 5:** Multivariate analysis of OS (INB—iBRAF/iMEK inhibitors or iBRAF alone, CHCT—chemotherapy, ITH—immunotherapy, Y = yes, N = no).

Additional Treatment		All	HR (Univariable)	HR (Multivariable)
INB (Y/N)	N	69 (78.4)	-	-
	Y	19 (21.6)	0.52 (0.24–1.11, *p* = 0.092)	0.66 (0.29–1.48, *p* = 0.315)
CHTH (Y/N)	N	55 (62.5)	-	-
	Y	33 (37.5)	2.02 (1.08–3.79, *p* = 0.028)	1.95 (1.00–3.80, *p* = 0.051)
ITH (Y/N)	N	53 (60.2)	-	-
	Y	35 (39.8)	0.59 (0.32–1.08, *p* = 0.085)	0.56 (0.30–1.03, *p* = 0.061)

**Table 6:** Multivariate analysis of PFS (INB—iBRAF/iMEK inhibitors or iBRAF alone, CHCT—chemotherapy, ITH—immunotherapy, Y = yes, N = no).

Additional Treatment		All	HR (Univariable)	HR (Multivariable)
INB (Y/N)	N	67 (77.9)	-	-
	Y	19 (22.1)	0.84 (0.46–1.53, *p* = 0.562)	1.15 (0.61–2.18, *p* = 0.670)
CHTH (Y/N)	N	54 (62.8)	-	-
	Y	32 (37.2)	2.39 (1.39–4.11, *p* = 0.002)	2.79 (1.56–5.00, *p* = 0.001)
ITH (Y/N)	N	53 (61.6)	-	-
	Y	33 (38.4)	0.76 (0.45–1.28, *p* = 0.303)	0.60 (0.34–1.05, *p* = 0.072)

**Table 7:** The multivariate analysis of OS in patients who achieved complete response after the first ECT procedure (INB—iBRAF/iMEK inhibitors or iBRAF alone, CHCT—chemotherapy, ITH—immunotherapy, Y = yes, N = no).

Additional Treatment		All	HR (Univariable)	HR (Multivariable)
INB (Y/N)	N	53 (75.7)	-	-
	Y	17 (24.3)	0.54 (0.24–1.23, *p* = 0.144)	0.75 (0.31–1.82, *p* = 0.528)
CHTH (Y/N)	N	44 (62.9)	-	-
	Y	26 (37.1)	2.45 (1.22–4.92, *p* = 0.012)	2.57 (1.20–5.52, *p* = 0.015)
ITH (Y/N)	N	43 (61.4)	-	-
	Y	27 (38.6)	0.46 (0.23–0.92, *p* = 0.029)	0.41 (0.20–0.84, *p* = 0.014)

**Table 8:** The multivariate analysis of CR ECT patients, who received systemic treatment (INB—iBRAF/iMEK inhibitors or iBRAF alone, CHCT—chemotherapy, ITH—immunotherapy, Y = yes, N = no).

Additional Treatment		All	HR (Univariable)	HR (Multivariable)
ITH (Y/N)	N	43 (62.3)	-	-
	Y	26 (37.7)	0.77 (0.44–1.35, *p* = 0.358)	0.58 (0.32–1.06, *p* = 0.079)
CHTH (Y/N)	N	43 (62.3)	-	-
	Y	26 (37.7)	2.61 (1.45–4.69, *p* = 0.001)	3.17 (1.65–6.09, *p* = 0.001)
INB (Y/N)	N	52 (75.4)	-	-
	Y	17 (24.6)	0.79 (0.41–1.52, *p* = 0.483)	1.21 (0.60–2.46, *p* = 0.591)

**Table 9:** PFS in patients treated with immunotherapy, or not (Y/N) after CR post ECT.

				95% Confidence Interval		
Levels	Time (Months)	Number at Risk	Number of Events	Survival	Lower	Upper
N	12	8	27	24.8 %	13.6 %	45.2 %
N	36	2	2	14.9 %	5.8 %	37.8 %
N	60	1	0	14.9 %	5.8 %	37.8 %
Y	12	10	16	38.5 %	23.7 %	62.5 %
Y	36	2	5	17.9 %	7.7 %	42.0 %
Y	60	2	0	17.9 %	7.7 %	42.0 %

## Data Availability

The data presented in this study are available upon request from the corresponding author. The raw data may need to be processed to remove sensitive personal information before releasing any data. Due to applicable law, the transfer of data may additionally require the approval of the Data Protection Officer.
